# Supplementary material for: Sympatric Populations of the Anopheles gambiae Complex in Southwest Burkina Faso Evolve Multiple Diverse Resistance Mechanisms in Response to Intense Selection Pressure with Pyrethroids
Source: Insects. 2022 Feb 28;13(3):247. doi: 10.3390/insects13030247 (PMC8955173; doi:10.3390/insects13030247)
Supplement: Supplementary file 1 [file insects-13-00247-s001.zip › Supplementary data/Supplementary Figure S1. (PBO simultaneous and sequential exposures).docx]

Supplementary file 2 Fig.1. PBO synergism results for three resistant anopheline strains with simultaneous and sequential exposures to PBO and permethrin (Perm) or deltamethrin (Delta). Mortality rates % (24) hours after exposure. Minimal sample size n=80. Error bars represent 95% binomial confidence intervals. Statistical differences between insecticide only and PBO + insecticide are indicated as **P*<0.05, or ns- not significant.
