# Supplementary material for: Sympatric Populations of the Anopheles gambiae Complex in Southwest Burkina Faso Evolve Multiple Diverse Resistance Mechanisms in Response to Intense Selection Pressure with Pyrethroids
Source: Insects. 2022 Feb 28;13(3):247. doi: 10.3390/insects13030247 (PMC8955173; doi:10.3390/insects13030247)

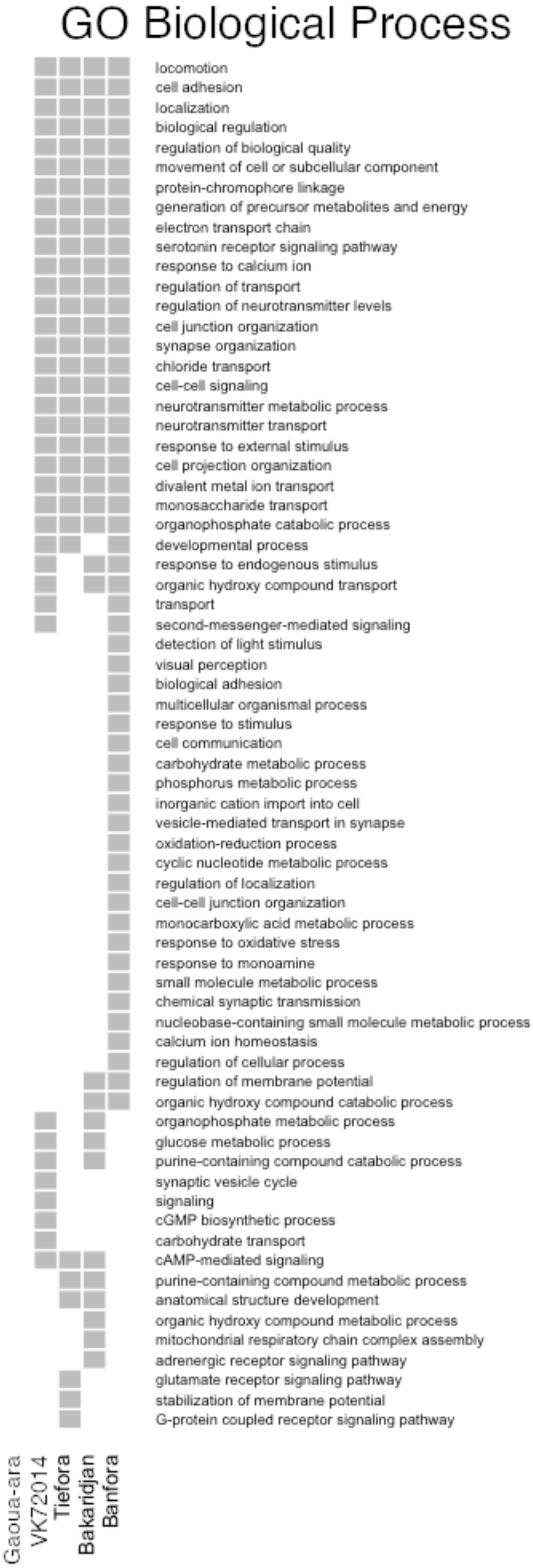
GO Biological Process
locomotion
cell adhesion
localization
biological regulation
regulation of biological quality
movement of cell or subcellular component
protein-chromophore linkage
generation of precursor metabolites and energy
electron transport chain
serotonin receptor signaling pathway
response to calcium ion
regulation of transport
regulation of neurotransmitter levels
cell junction organization
synapse organization
chloride transport
cell-cell signaling
neurotransmitter metabolic process
neurotransmitter transport
response to external stimulus
cell projection organization
divalent metal ion transport
monosaccharide transport
organophosphate catabolic process
developmental process
response to endogenous stimulus
organic hydroxy compound transport
transport
second-messenger-mediated signaling
detection of light stimulus
visual perception
biological adhesion
multicellular organismal process
response to stimulus
cell communication
carbohydrate metabolic process
phosphorus metabolic process
inorganic cation import into cell
vesicle-mediated transport in synapse
oxidation-reduction process
cyclic nucleotide metabolic process
regulation of localization
cell-cell junction organization
monocarboxylic acid metabolic process
response to oxidative stress
response to monoamine
small molecule metabolic process
chemical synaptic transmission
nucleobase-containing small molecule metabolic process
calcium ion homeostasis
regulation of cellular process
regulation of membrane potential
organic hydroxy compound catabolic process
organophosphate metabolic process
glucose metabolic process
purine-containing compound catabolic process
synaptic vesicle cycle
signaling
cGMP biosynthetic process
carbohydrate transport
cAMP-mediated signaling
purine-containing compound metabolic process
anatomical structure development
organic hydroxy compound metabolic process
mitochondrial respiratory chain complex assembly
adrenergic receptor signaling pathway
glutamate receptor signaling pathway
stabilization of membrane potential
G-protein coupled receptor signaling pathway
Gaoua-ara
VK72014
Tiefora
Bakaridjan
Banfora

GO Cellular Component

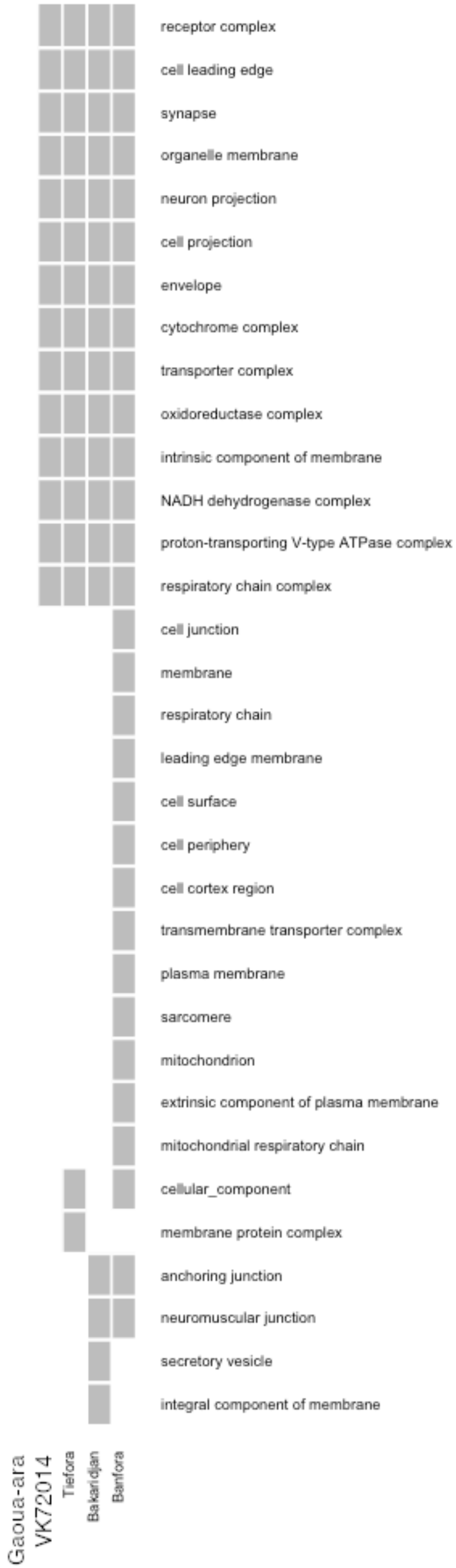

GO Molecular Function

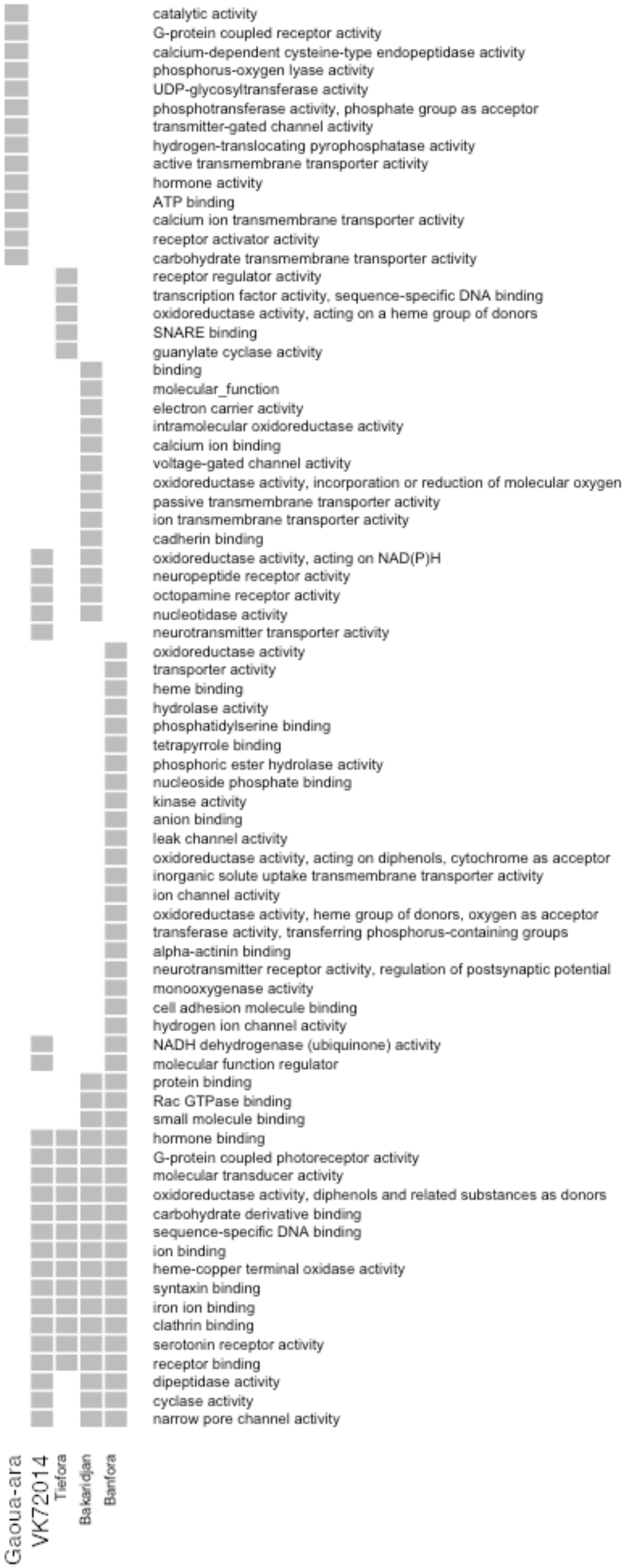

Other Pathways

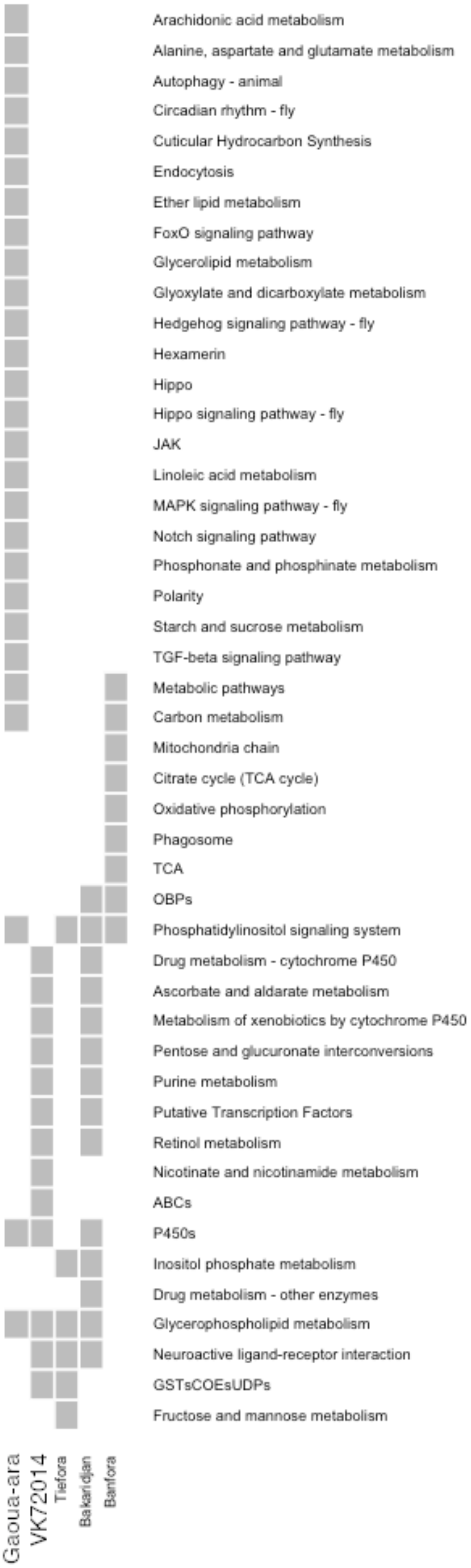

Supplement: Supplementary file 1 [file insects-13-00247-s001.zip › Supplementary data/Supplementary Figure S3 Up enrichment figure.pdf]
