# Supplementary material for: Sympatric Populations of the Anopheles gambiae Complex in Southwest Burkina Faso Evolve Multiple Diverse Resistance Mechanisms in Response to Intense Selection Pressure with Pyrethroids
Source: Insects. 2022 Feb 28;13(3):247. doi: 10.3390/insects13030247 (PMC8955173; doi:10.3390/insects13030247)

Other Pathways

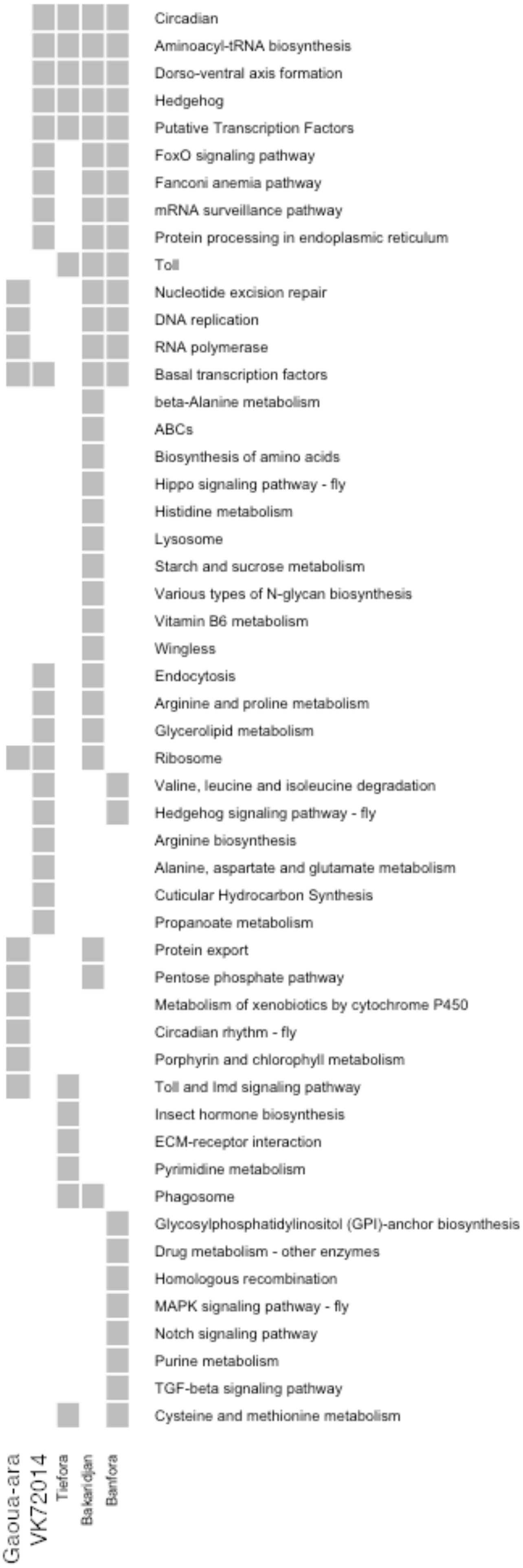

GO Biological Process

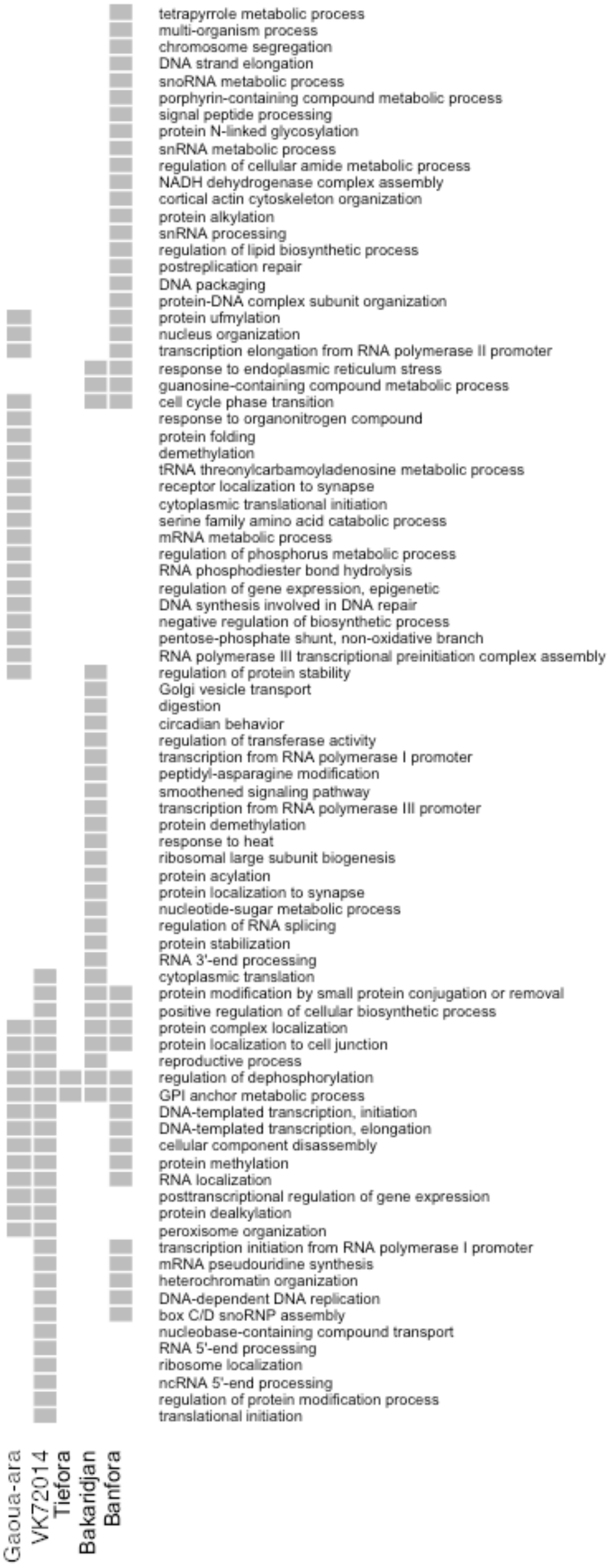

GO Cellular Component

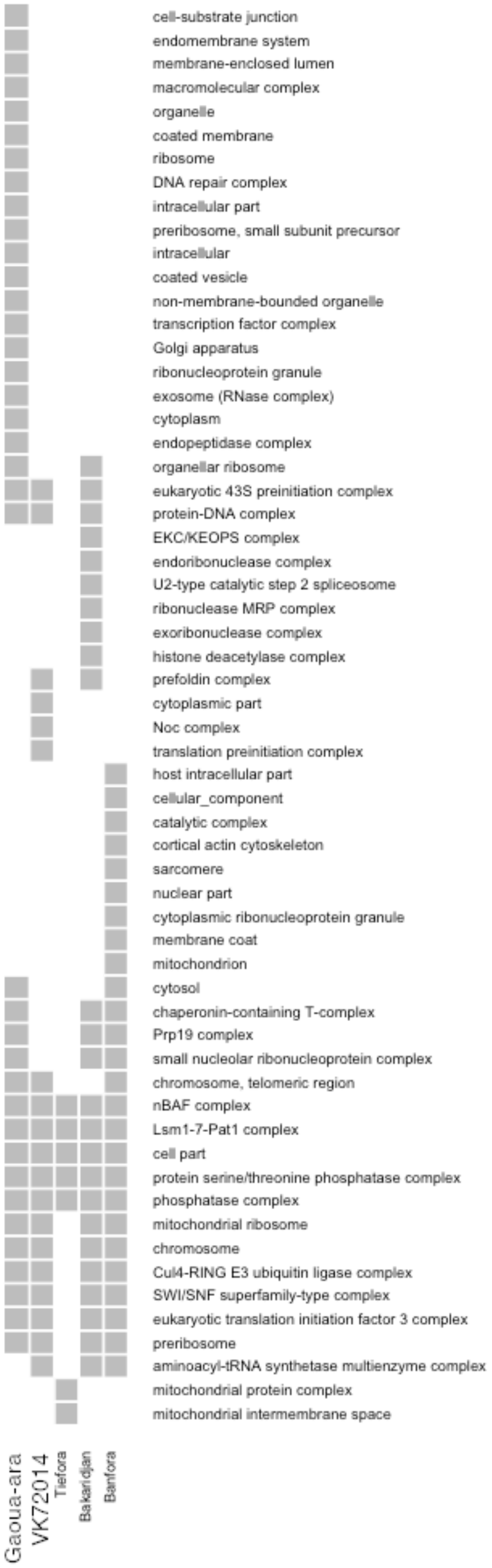

GO Molecular Function

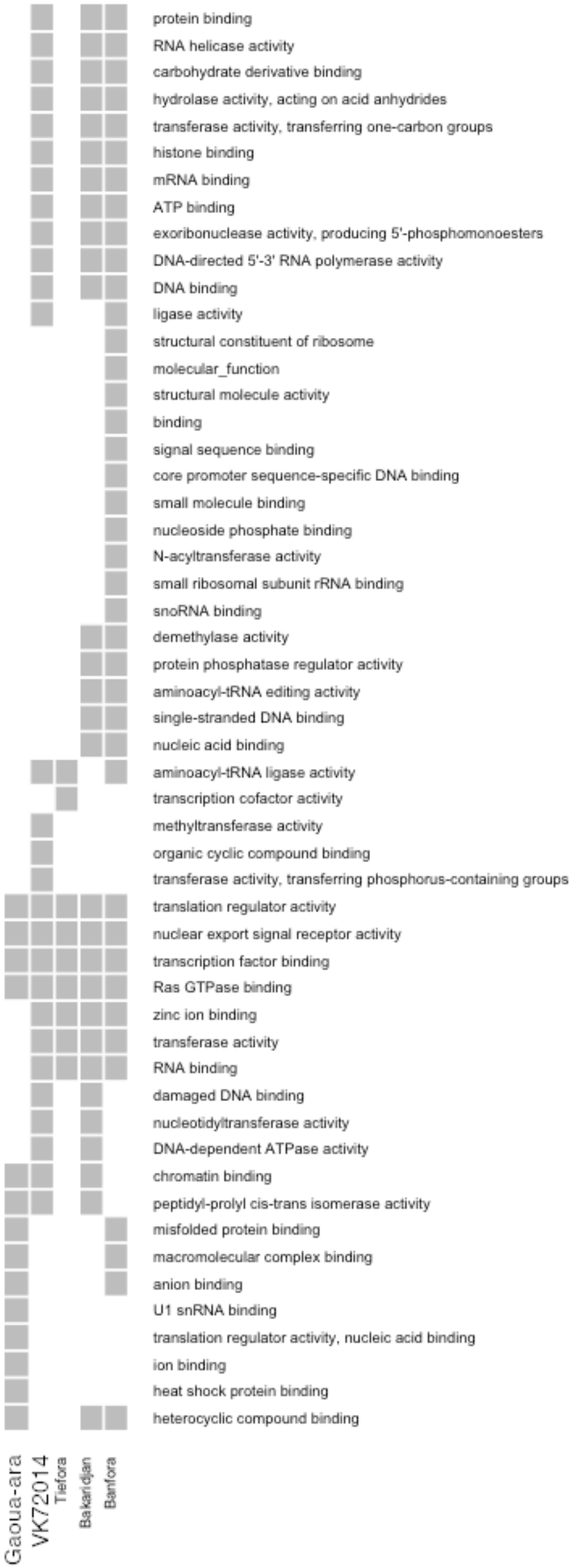

Supplement: Supplementary file 1 [file insects-13-00247-s001.zip › Supplementary data/Supplementary Figure S4 Down enrichment figure.pdf]
