# Supplementary figures and images for: Sympatric Populations of the Anopheles gambiae Complex in Southwest Burkina Faso Evolve Multiple Diverse Resistance Mechanisms in Response to Intense Selection Pressure with Pyrethroids
Source: Insects. 2022 Feb 28;13(3):247. doi: 10.3390/insects13030247 (PMC8955173; doi:10.3390/insects13030247)

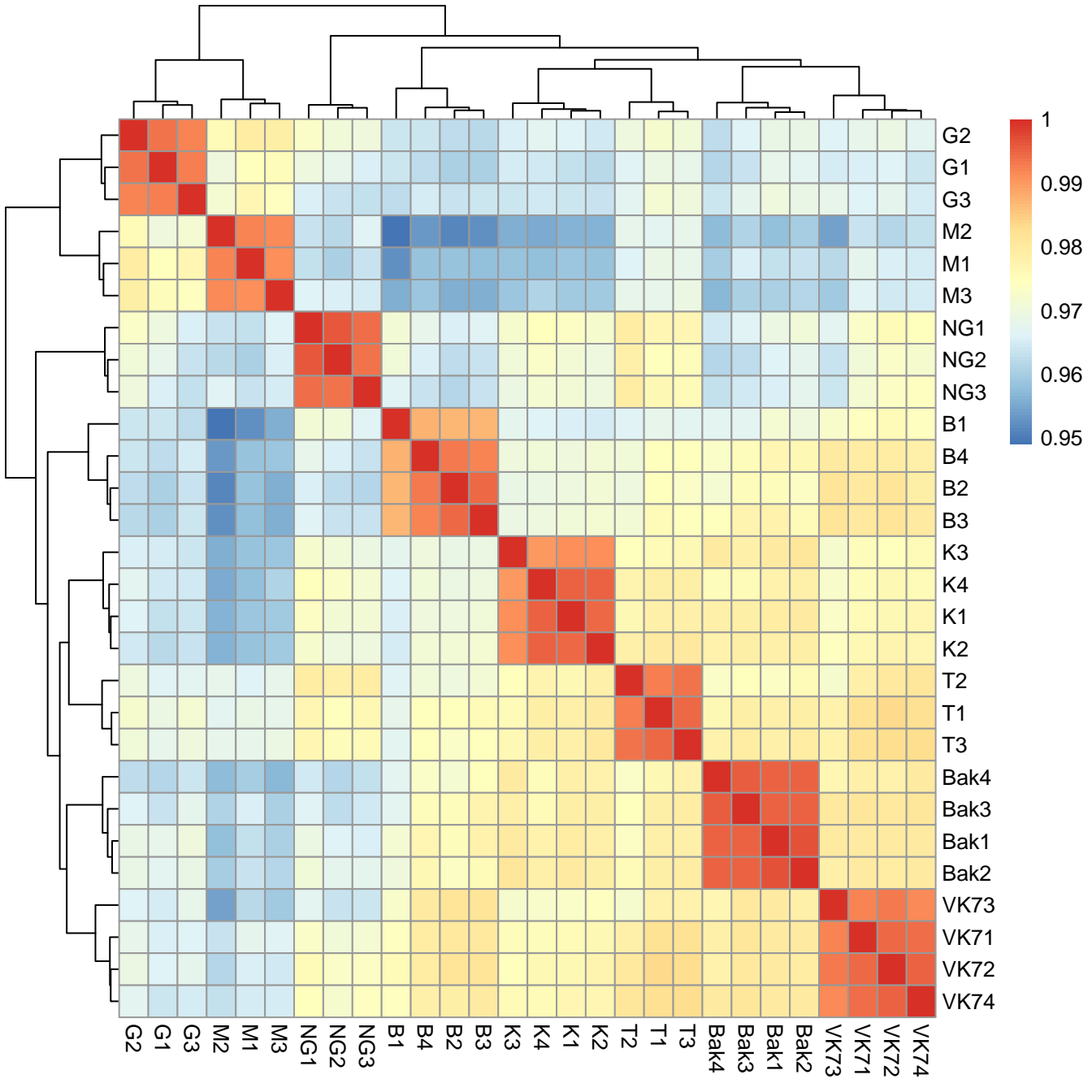

Supplement: Supplementary file 1 [file insects-13-00247-s001.zip › Supplementary data/Supplementary Figure S2 Correlation_plot.pdf]

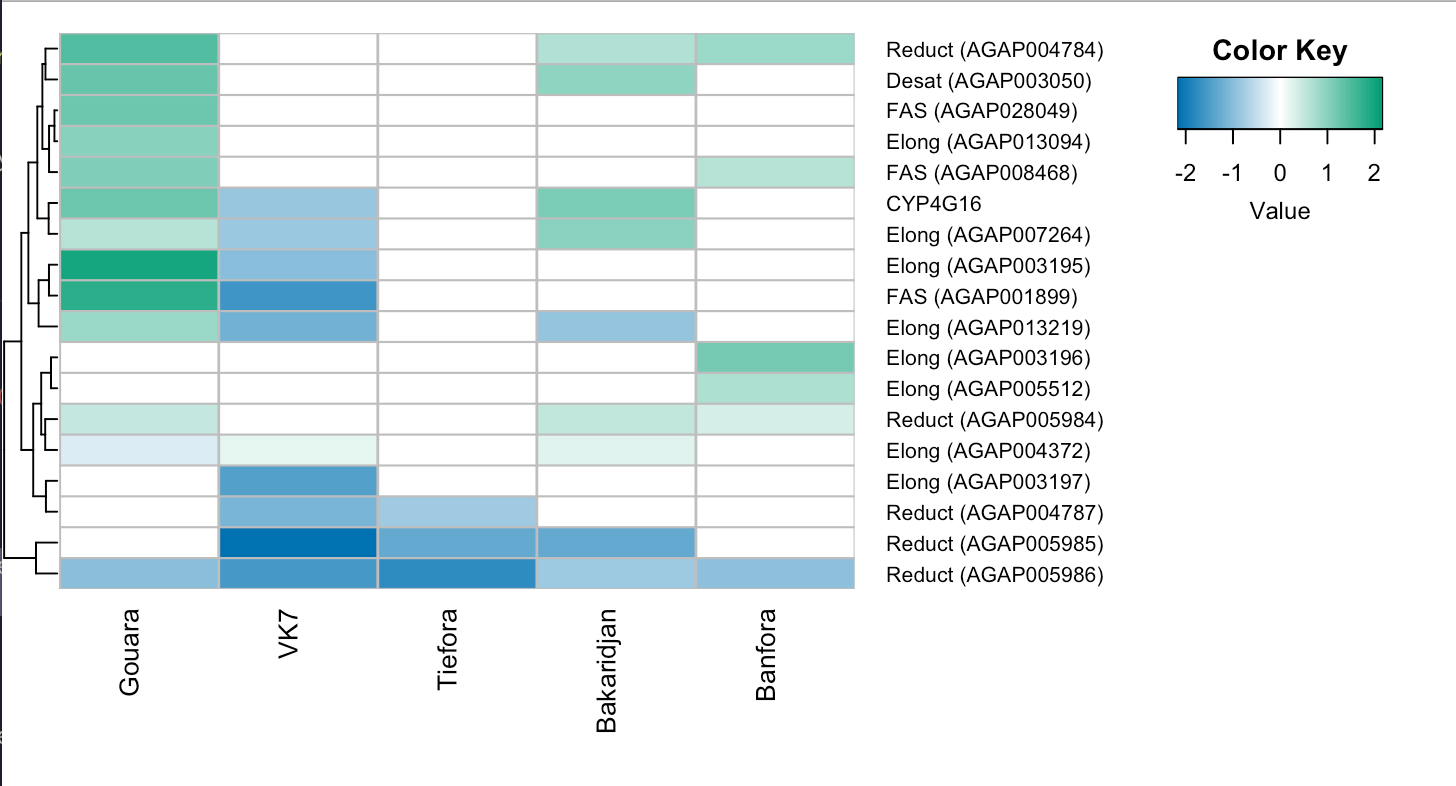

Supplement: Supplementary file 1 [file insects-13-00247-s001.zip › Supplementary data/Supplementary Figure S5 Cuticular Pathway key.png]
